# Editorial: Radioresistance in breast cancer

**DOI:** 10.3389/fonc.2024.1514173

**Published:** 2024-11-13

**Authors:** Zhuo-Fei Bi, San-Gang Wu, Zhe-Sheng Chen

**Affiliations:** ^1^ Department of Radiation Oncology, Sun Yat-Sen Memorial Hospital, Sun Yat-Sen University, Guangzhou, China; ^2^ Guangdong Provincial Key Laboratory of Malignant Tumor Epigenetics and Gene Regulation, Medical Research Centre, Sun Yat-Sen Memorial Hospital, Sun Yat-Sen University, Guangzhou, China; ^3^ Department of Radiation Oncology, Xiamen Cancer Center, Xiamen Key Laboratory of Radiation Oncology, the First Affiliated Hospital of Xiamen University, School of Medicine, Xiamen University, Xiamen, China; ^4^ Department of Pharmaceutical Sciences, College of Pharmacy and Health Sciences, St. John’s University, Queens, NY, United States

**Keywords:** breast cancer, radiotherapy, radioresistance, target volume, radiation dose

Breast cancer is the most commonly diagnosed cancer in women globally (1). The estimated numbers of breast cancer cases account for 32% of all new diagnoses for women in the United States in 2024 (2). Radiotherapy is an important component of breast cancer therapy. Adjuvant radiotherapy is the standard of care for patients receiving breast-conserving surgery, which can significantly reduce locoregional recurrence and the breast cancer death rate (3). Regional node radiotherapy in early breast cancer patients significantly reduced breast cancer mortality and all-cause mortality in trials done in recent years (4). However, the development of radioresistance remains a major challenge in improving outcomes for breast cancer patients who receive radiotherapy (5).

This Research Topic aims to explore the mechanisms of radioresistance and find promising predictive and therapeutic methods for radioresistant breast cancer patients. 8 articles were accepted.

Screening of radioresistant breast cancer patients is important for improving the effect of radiotherapy. In this topic, Zhang et al. proposed a novel radiotherapy response classification system based upon molecular profiles for estimating radiosensitivity for individual breast cancer patients and elucidated a methodological advancement for the synergy of radiotherapy with immune checkpoint blockade (ICB). This study classified breast cancer patients to radiotherapy-sensitive and radiotherapy-resistant clusters by quantitative RT-PCR analyses of differentially expressed radiotherapy response-related genes (DERRGs). Then, the Riskscore was proposed to improve patient prognosis. The high Riskscore samples had lower radiotherapeutic response and stronger DNA damage repair as well as poor anticancer immunity. Zheng et al. found that a higher Nottingham prognostic index (NPI) score is an independent risk factor for predicting locoregional recurrence in breast cancer. Two nomogram prediction models related to radioresistance were constructed in this study. Six significant variables were identified for the models, including age, body mass index (BMI), TNM stage, NPI, vascular invasion, and perineural invasion. The nomogram prediction model based on the NPI has undergone internal validation and has been found to have good discrimination and calibration. The two studies highlight the importance of identifying radioresistant breast cancer patients through different clinical variables and special radiotherapy response-related genes.

The de-escalation of radiotherapy for selected breast cancer patients is also important for overcoming radioresistance. Because prolonged radiation therapy is more likely to induce secondary radioresistance. Liu et al. conducted a retrospective analysis and found that omitting regional lymph node irradiation may be a safe option in T1-2N1 HER2-overexpressing by breast cancer patients receiving standardized anti-HER2 targeted therapy, particularly in ER-positive or lymph vascular invasion status (LVI)-negative subgroups.

The strategies of de-escalated radiotherapy in target volume are also researched for breast cancer patients. The European Society for Radiotherapy and Oncology–Advisory Committee in Radiation Oncology Practice (ESTRO-ACROP) updated a new target volume delineation guideline for postmastectomy radiotherapy after implant-based reconstruction. Park et al. aimed to evaluate the impact on breast complications with the new guideline compared to the conventional guidelines, and found that the new ESTRO-ACROP target volume guideline did not demonstrate significant differences in major or any breast complications compared to the conventional guidelines. It showed a tendency to reduce complication risks by the volume de-escalation of radiotherapy.

Tumor volume and radiation dose are predictors of response and can be used to guide the decision-making for patients with radioresistant brain metastases (6). Stereotactic radiosurgery offers a benefit over whole-brain radiation, which is attributed to the reduction of radioresistance and neurotoxicity (7). Li et al. conducted a retrospective analysis to study the role of brain radiotherapy in HER2-positive breast cancer with brain metastases. Because it is considered that with the development of HER2-targeted therapies, brain radiotherapy can be delayed, and stereotactic radiosurgery (SRS) can be used in more patients. The authors concluded that HER2-positive breast cancer with brain metastases still faces significant challenges, particularly those with high intracranial tumor burden, which correlates with poorer outcomes and higher incidence of leptomeningeal metastasis. Larger tumors necessitated more comprehensive treatment approaches, such as whole-brain radiation therapy (WBRT) and SRS.

And the target tumor volume and dose of intraoperative radiotherapy (IORT) are also much smaller than conventional radiation. Bhimani et al. demonstrate the successful use of IORT for breast-conserving surgery in patients with bilateral breast cancer (BBC) by their case series. The authors concluded that IORT may serve as an excellent patient-centered alternative for BBC, considering the benefits of improved patient compliance and a reduced number of multiple visits.

HER2-targeting monoclonal antibodies can be combined with radiation in breast cancer, apparently with no excess toxicities (8). The developments of antibody-drug conjugate (ADC) offer an opportunity for the use of radiotherapy in refractory breast cancer. Gui et al. conducted a disproportionality analysis using spontaneous reports in the FDA adverse event reporting system to assess the real-world safety concerns of Sacituzumab Govitecan (SG). SG is a novel ADC that is a trophoblastic cell surface antigen-2 (TROP-2) directed antibody conjugated to the active metabolite of irinotecan, SN-38 (govitecan). This study provides crucial real-world safety data on SG, complementing existing clinical trial information.

The manuscript by Arnold et al. is a remarkable review of this Research Topic: Innovative Therapeutic Strategies to Overcome Radioresistance in Breast Cancer. Arnold et al. concluded that in the context of combating radioresistance in breast cancer, potential targets of interest include long non-coding RNAs (lncRNAs), micro RNAs (miRNAs), and their associated signaling pathways, along with other signal transduction routes amenable to pharmacological intervention. Furthermore, technical, and methodological innovations, such as the integration of hyperthermia or nanoparticles with radiotherapy, have the potential to enhance treatment responses in patients with radioresistant breast cancer.

In summary, the developments of novel predictive models for identifying radioresistant patients and the strategies of de-escalated locoregional radiotherapy in selected patients are important for overcoming radioresistance of breast cancer. Continued basic and clinical research for radioresistant breast cancer is of vital importance to improve the prognosis of patients.
